# Water quality measurements in Buzzards Bay by the Buzzards Bay Coalition *Baywatchers* Program from 1992 to 2018

**DOI:** 10.1038/s41597-021-00856-4

**Published:** 2021-03-05

**Authors:** Rachel W. Jakuba, Tony Williams, Christopher Neill, Joseph E. Costa, Richard McHorney, Lindsay Scott, Brian L. Howes, Hugh Ducklow, Matthew Erickson, Mark Rasmussen

**Affiliations:** 1 Buzzards Bay Coalition, 114 Front St, New Bedford, MA 02740 USA; 2 Marine Biological Laboratory, 7 MBL Street, Woods Hole, MA 02543 USA; 3 Woodwell Climate Research Center, 149 Woods Hole Road, Falmouth, MA 02540 USA; 4 Buzzards Bay National Estuary Program, Massachusetts Office of Coastal Zone Management, 81-B County Rd Suite E, Mattapoisett, MA 02739 USA; 5 School for Marine Science and Technology, University of Massachusetts, Dartmouth, 706 South Rodney French Boulevard, New Bedford, MA 02744 USA; 6 Present Address: Lamont-Doherty Earth Observatory, Columbia University, 61 Route 9W, Palisades, NY 10964 USA; 7 Present Address: General Dynamics Information Technology, 6361 Walker Lane, Suite 300, Alexandria, Virginia 22310 USA

**Keywords:** Marine biology, Element cycles, Marine chemistry

## Abstract

The Buzzards Bay Coalition’s Baywatchers Monitoring Program (*Baywatchers*) collected summertime water quality information at more than 150 stations around Buzzards Bay, Massachusetts from 1992 to 2018. *Baywatchers* documents nutrient-related water quality and the effects of nitrogen pollution. The large majority of stations are located in sub-estuaries of the main Bay, although stations in central Buzzards Bay and Vineyard Sound were added beginning in 2007. Measurements include temperature, salinity, Secchi depth and concentrations of dissolved oxygen, ammonium, nitrate + nitrite, total dissolved nitrogen, particulate organic nitrogen, particulate organic carbon, ortho-phosphate, chlorophyll *a*, pheophytin *a*, and in lower salinity waters, total phosphorus and dissolved organic carbon. The *Baywatchers* dataset provides a long-term record of the water quality of Buzzards Bay and its sub-estuaries. The data have been used to identify impaired waters, evaluate discharge permits, support the development of nitrogen total maximum daily loads, develop strategies for reducing nitrogen inputs, and increase public awareness and generate support for management actions to control nutrient pollution and improve water quality.

## Abstract (summary)

**Table:** 

Measurement(s)	temperature of water • dioxygen dissolved in marine water • salinity of water • water clarity • concentration of ammonium in water • concentration of nitrate plus nitrite in water • concentration of phosphate in water • concentration of nitrogen atom in water • concentration of particulate organic carbon in water • concentration of total phosphorus in water • concentration of dissolved organic carbon in water • chlorophyll a • pheophytin a • concentration of particulate nitrogen in water
Technology Type(s)	Thermometer Device • titration • hydrometer • Secchi disk • spectrophotometry • chemical digestion followed by spectrophotometry • carbon, hydrogen and nitrogen elemental analysis • catalytic and persulphate oxidation • fluorometry
Factor Type(s)	year of data collection
Sample Characteristic - Environment	coastal sea water • bay • estuary
Sample Characteristic - Location	Buzzards Bay

Machine-accessible metadata file describing the reported data: 10.6084/m9.figshare.13363436

## Background & Summary

Nutrient pollution is an important driver of water quality degradation in the U.S. and around the world^1–4^. In coastal waters, nitrogen inputs are linked to greater phytoplankton growth, reduced water clarity, hypoxia, and declines in seagrass coverage, fish, and shellfish populations^5–7^. Because the magnitude and effects of nitrogen pollution vary with the characteristics of watersheds and receiving waters, water quality responses to nitrogen inputs—and potential strategies to mitigate those inputs—are site specific. Long-term records of coastal water quality are essential to document the effectiveness of management action, consequences of inaction, and related water quality drivers, like climate change.

Buzzards Bay is a 650 km^2^ estuary in Southeastern Massachusetts with a 1,123 km^2^ watershed that encompasses all or parts of 21 municipalities in two states and has a current year-round population of 250,000^8^. Buzzards Bay’s coastline spans 563 km and includes shallow river- and groundwater-fed sub-estuaries. The central Bay is 45 km long, averages 13 km wide, and has a mean depth of 11 m. Sub-estuaries range from several hectares to 19 km^2^. The Bay’s shoreline includes 21 km of heavily-used public beaches.

Beginning in 1984, Buzzards Bay received federal recognition and funding because toxic chemicals, bacteria, and nutrients threatened its economic and natural resources. In 1991, the Buzzards Bay National Estuary Program (NEP) developed a Comprehensive Conservation and Management Plan^9^ to protect and restore the estuary. Because of losses of eelgrass habitat^10^ and other impairments in Buzzards Bay, nitrogen pollution was one of the primary focuses of the NEP. The NEP and scientists from local research institutions designed a water quality monitoring strategy to support management action in Buzzards Bay^11^. The NEP identified engaging volunteer samplers as a cost-effective strategy for sampling numerous sub-estuaries, with an added benefit of raising local awareness of nitrogen pollution. The resulting monitoring program – *Baywatchers –* was modelled after other successful volunteer monitoring programs^12,13^ that combine field measurements and sample collections by staff and trained citizens (monitors) with water sample analysis by a research laboratory.

Monitors measure temperature, salinity, dissolved oxygen (D.O.), and Secchi depth and record weather and tide observations about every five days from late May to mid-September. Twice in July and twice in August, monitors collect whole water and filtered water samples, then transport them to a research laboratory for analysis of salinity, ammonium (NH_4_^+^), nitrate + nitrite (NO_3_^−^ + NO_2_^−^), soluble reactive phosphorus (PO_4_^3−^), total dissolved nitrogen (TDN), particulate organic nitrogen (PON), particulate organic carbon (POC), chlorophyll *a* (Chl *a*) and pheophytin *a* (Pheo). Total phosphorus (TP) and dissolved organic carbon (DOC, beginning in 2009) are measured on a subset of fresh water or very low salinity stations. Sample collection and analysis follow a Quality Assurance Project Plan approved by the Massachusetts Department of Environmental Protection and the U.S. Environmental Protection Agency^14^.

**Fig. 1:**
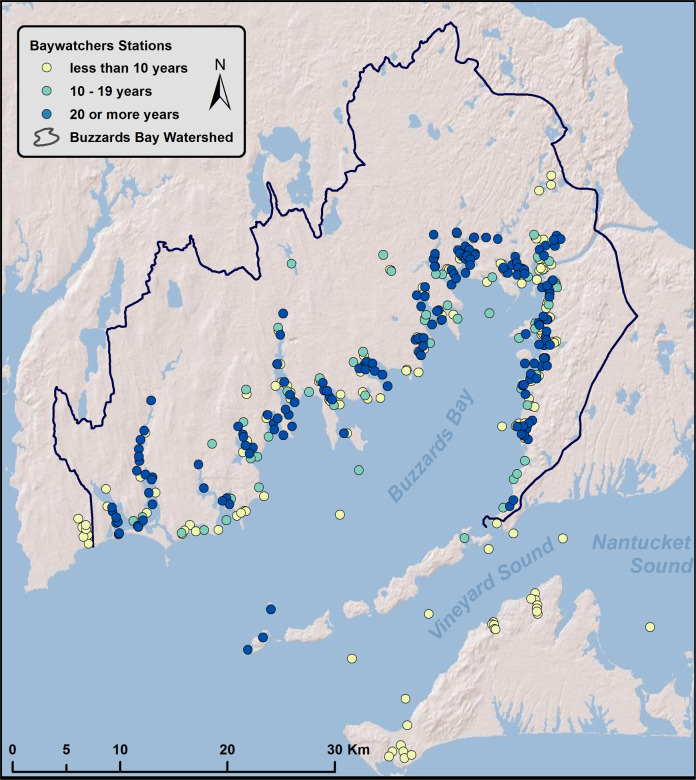
Location of water quality sampling stations. Circle color indicates the number of years that data has been collected at that station. Black line indicates the Buzzards Bay watershed.

**Fig. 2:**
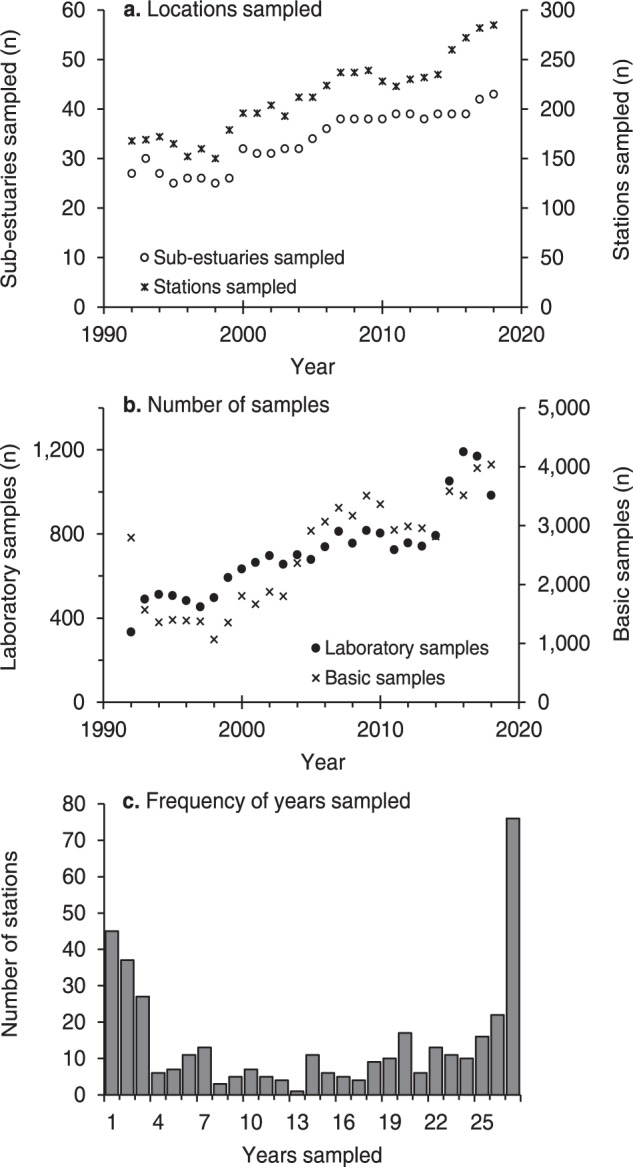
*Baywatchers* sampling effort over time: (**a**) number of sub-estuaries and stations sampled, (**b**) number of laboratory water quality samples (NH_4_^+^, NO_3_^-^ + NO_2_^-^, PO_4_^3-^, TDN, PON, POC, Chl *a*, Pheo) and basic samples (dissolved oxygen, temperature, salinity, Secchi depth) collected, and (**c**) frequency  that stations were sampled during the program’s 27 year history.

Sampling locations are primarily near shore (Fig. 1) with many located at public docks and piers to provide easy and repeated access. More than 150 sites are monitored in a typical year (Fig. 2a). The *Baywatchers* dataset contains approximately 19,000 records of NH_4_^+^, NO_3_^−^ + NO_2_^−^, PO_4_^3−^, TDN, PON, POC, Chl *a* and Pheo (Fig. 2b, average = 712 samples per year) and over 72,000 records of temperature, salinity, and D.O. (Fig. 2b, average = 2,513 samples per year) made over 27 years. Each year, temperature, salinity, D.O., and Secchi depth were measured at between 60 and 215 stations (average = 130 stations). Samples for laboratory analysis were collected at between 89 and 230 stations (average = 155 stations). Some stations have been added or dropped over time, but approximately half of the stations have been sampled for 17 or more years and 76 stations have been sampled for 27 years (Fig. 2c).

The data have been used by local, state, and federal managers to identify impaired waters, evaluate discharge permits, to support the development of nitrogen total maximum daily loads, and track pollution reduction. Researchers have used the data to evaluate estuarine health metrics^15^, evaluate climate change impacts on water quality^16^, and compare nitrogen loading and water quality^17^. The results have also been used to build public support for municipal bylaws and regulations that protect water quality.

## Methods

### Sampling stations

*Baywatchers* sampling stations were generally concentrated in the upper half of estuaries and major sub-estuaries to better characterize water quality changes over time. The program has grown over time and expanded to include additional stations within sub-estuaries as well as adding additional sub-estuaries. In 2012, stations were added in Vineyard Sound, which adjoins Buzzards Bay, and in 2017, sampling began at additional stations in the coastal ponds connected to Vineyard Sound.

We assign each station a unique identification code (station ID) and data are integrated into geographic information systems. Station maps are given to monitors (historically using ArcView GIS overlaid on scanned U.S.G.S. quadrangle maps, but more recently, using Google Earth to produce the maps overlaid on aerial images). In a small number of cases, the location of a monitoring site has varied slightly over time—for example, nutrient samples were collected in the Agawam River in a rowboat 250 feet from shore from 1998 to 2007 (station AG2A), but have been collected from a nearby dock since 2008 (station AG2). When a monitoring site’s location has moved, it is given a unique station ID in the database (i.e., AG2 vs AG2A in the example above).

### Field sampling

Sampling occurs from late May to September to document conditions when biological activity is highest. Field water sampling is separated into “basic” sampling and “laboratory” sampling days. On all sampling dates, water temperature, salinity, Secchi depth, and total depth are measured in the field. Monitors record these results on hard copy datasheets along with the station ID, sampling date, collection time, name of person sampling, and name of the sub-estuary.

**Fig. 3:**
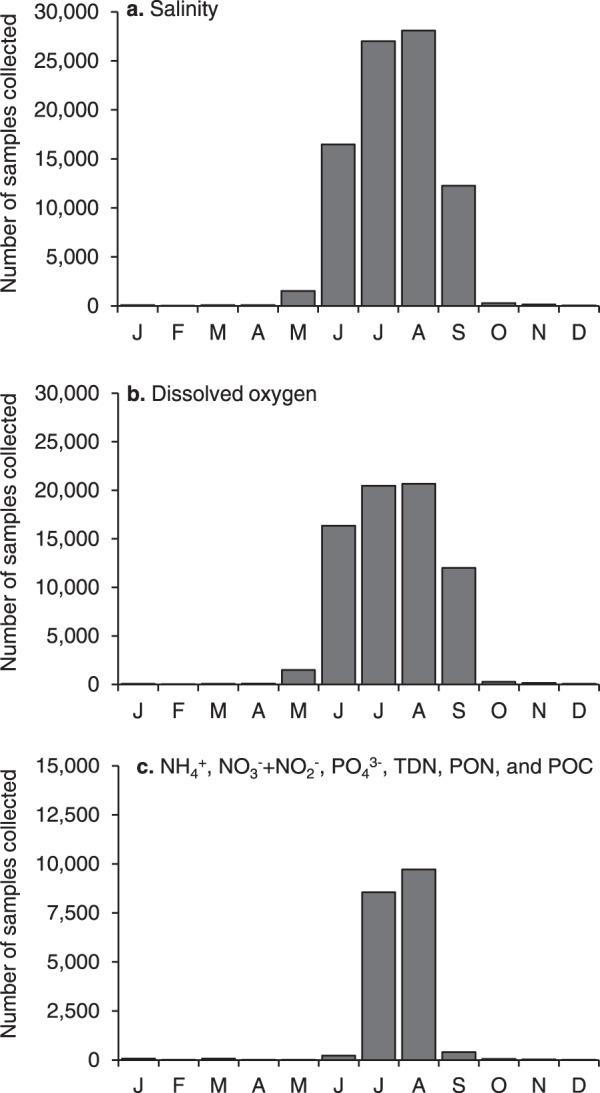
Frequency of *Baywatchers* samples collected by month between 1992 and 2018. Note difference in y-axis scale for panel c.

On basic sampling days, D.O. is measured in the early morning (between 6:00 and 9:00 am) to capture typical daily minimum oxygen concentrations before peak daytime photosynthetic oxygen production. Basic sampling occurs on a schedule roughly every five days between late May and mid-September. On laboratory sampling days, oxygen measurements are only made if the monitor has a water quality sonde, as the focus is the collection of samples for laboratory analysis of NH_4_^+^, NO_3_^−^ + NO_2_^−^, PO_4_^3−^, TDN, PON, POC, Chl *a*, and Pheo. At designated fresh water and low salinity stations, TP and DOC are also measured. Laboratory sampling occurs on four scheduled days each summer (2 in July, 2 in August) during the last three hours of an outgoing tide when concentrations of solutes in estuarine water are expected to most strongly reflect the influence of watershed inputs. While the vast majority of observations were made between late May and mid-September (Fig. 3), some additional basic and laboratory sampling occurred at other times of the year when short-term projects provided opportunities for expanded sampling.

The Buzzards Bay Coalition pairs some nearby basic sampling stations with laboratory sampling stations for analysis of sub-estuary water quality. These station pairs have station IDs that end in either the suffix X or N to indicate that they are sampled on basic or laboratory sampling days, respectively.

#### Basic sampling procedures

Water samples are collected for water temperature, salinity, and D.O. from near the bottom of the water column (0.3 m above the bottom). Where the water column is deeper than 1.2 m, a sample near the surface (0.15 m depth) is also collected to provide information on potential water column stratification. The depth of 0.15 m below the surface prevents entrainment of floating particles and overlying air into the sample bottles. Sampling 0.3 m above the bottom prevents resuspension and capture of bottom sediments by the sampling apparatus.

Water samples for temperature, salinity, and D.O. are collected either with a steel sampling pole or measured *in situ* with water quality sondes (YSI models 600XL, 600XLM, 6600, EXO2, ProDSS). Sampling poles are 1.5 or 3 m long and marked in 5 to 10 cm depth increments. Sampling poles have 1 L and 0.5 L plastic (HDPE) bottles with rubber stopper closures connected to strings. Poles are lowered to the appropriate depth and then bottles are opened by pulling the strings, first the 0.5 L bottle is opened, followed by the 1 L bottle. D.O. is measured from the 0.5 L bottle, so it is opened first to prevent entrainment of air bubbles into the D.O. sample. Temperature and salinity are measured from the 1 L bottle. Water temperature is measured directly in the 1 L bottle using a thermometer that is calibrated annually. Monitors have primarily used analog thermometers; however, some digital thermometers have been used since 2016. Salinity is measured by then transferring 0.5 L of sample from the 1 L bottle to a 0.5 L graduated cylinder. Specific gravity is measured using a hydrometer that is calibrated annually. Temperature is measured in the graduated cylinder and salinity is determined from a table of specific gravity and temperature.

The 0.5 L bottle has been modified with plastic fittings and tubing at the bottom so that water can be extracted from the bottom of the bottle. Water is siphoned through the tubing to the bottom of a glass-stoppered bottle, overflowing the glass bottle until the 0.5 L bottle is only one-quarter full. Monitors measure D.O. in the glass bottle directly in the field using a modified Winkler titration (Hach Test Kit, Model OX-2P). Briefly, pre-weighed aliquots of manganese sulphate and a lithium hydroxide monohydrate/potassium iodide mixture are added to the sample bottle, which is stoppered and vigorously shaken. The resultant floc is allowed to settle, then reshaken and settled again, before the addition of a pre-weighed aliquot of a sodium phosphate dibasic/sodium sulphate/citric acid mixture. The sample is shaken until this dissolves and the sample is clear, then an aliquot of sample is measured into a separate vial. The sample aliquot is titrated drop-wise using a sodium thiosulphate standard until the sample becomes colorless. The D.O. concentration (mg L^−1^) is calculated from the number of titration drops.

Secchi depth is recorded by lowering a Secchi disk into water slowly from the shady side of a boat, dock or pier until it just disappears from view. It is then raised and lowered slightly to ensure the proper average depth of disappearance. If the Secchi disk hits the bottom before it disappears, no Secchi depth value is recorded. Total depth is determined when slack is felt in the measuring tape of the Secchi disk.

On basic sampling days, monitors also record the tidal direction (ebb or flood) and the time of the nearest low tide according to the Eldridge Tide and Pilot Book, wave conditions according to the Beaufort scale, and weather status (based on eight potential choices: cloudless, partly cloudy, overcast, fog/haze, drizzle, intermittent rain, rain, snow). Precipitation in the previous 24 hours is noted as either none, light, or heavy. Wind direction is also recorded. All data are recorded on a paper data sheet.

Approximately 20,000 of the temperature, salinity, and D.O. and roughly 5,000 of the pH and Chl *a* measurements were made *in situ* using water quality sondes. The majority of these measurements were made since 2000 (YSI models 600XL, 600XLM, 6600, EXO2, ProDSS), though a few were made in the early years of the program (YSI model 51B). The sonde measurements are made following the manufacturers’ standard operating procedures. Instruments are calibrated for temperature, salinity, D.O., pH and Chl *a* at the beginning of each sampling season. The instrument D.O. calibration is checked prior to each sampling day and re-calibrated in the field if necessary.

#### Laboratory sampling procedures

On laboratory sampling days, water samples for analysis of dissolved and particulate constituents are primarily collected from near the surface (0.15 m), though a small portion have been collected from near the bottom (0.3 m above bottom). Bottom water samples were generally collected near where there is significant freshwater input that could cause water column stratification. Station ID, water temperature, salinity, Secchi depth, total depth, sample depth, and collection time are recorded in the field on hard copy data sheets. Monitors with sondes also record D.O.

Samples are collected directly into 1 L acid-washed plastic HDPE bottles either by hand or using the sampling pole. All bottles used in water collection were acid washed by the analytical laboratory. Samples bottles are rinsed once with sample prior to filling with the sample. When using the sampling pole, sample bottles are attached and removed using hose clamps.

Monitors filter 60 mL of sample from the 1 L bottle using a 0.2 μm cellulose acetate membrane filter. Filters are first rinsed with 30 mL of sample, which is discarded. A subsequent 30 mL of filtered sample is used to rinse the 60 mL sampling bottle before an additional 60 mL are filtered directly into the 60 mL bottle that was previously acid washed by the analytical laboratory. The remaining unfiltered sample and the 60 mL filtered sample are stored in coolers with ice packs and delivered on the day they are collected to the analytical laboratory. A new membrane filter is used for each sample and the filter holder and syringes are rinsed with tap water after a sample is filtered.

### Laboratory analyses

Laboratory analyses were conducted under the supervision of B. Howes at the Woods Hole Oceanographic Institution (1992–1997) and at the University of Massachusetts, Dartmouth (1998–2007), and under H. Ducklow (2008–2012) and C. Neill (2013–2018) at the Marine Biological Laboratory. The methods and instruments described below are those currently used. In some cases, the instruments used have changed, but there has been a significant effort to maintain consistency over the lifetime of the program and to intercalibrate methods/instruments when a change has been made.

Laboratory analyses are designed to accommodate the samples that range from fresh water to nearly full strength seawater and analyte concentrations that range from at or below the detection limit for a method to 1,000 times the detection limit in some cases. Laboratory staff used aliquots of the 60 mL field-filtered sample to perform the dissolved analyses (NH_4_^+^, NO_3_^−^ + NO_2_^−^, PO_4_^3−^, TDN). The filtration for the particulate analyses (Chl *a*, Pheo, PON, POC) was performed by laboratory staff using water from the 1 L dark sample bottle.

NH_4_^+^ is measured colorimetrically by the indophenol-hypochlorite method^18^. Analyses are conducted in pre-reacted test tubes to reduce blank corrections and absorbance is read on a Cary spectrophotometer with an automatic sipper attachment. NH_4_^+^ is analysed on the day samples are collected.

NO_3_^−^ + NO_2_^−^ is measured colorimetrically after cadmium reduction^19^ on a Lachat flow injection analyser (Hach, Loveland, CO). The method was modified for flow injection analysis by Lachat Instruments^20^. Prior to analysis, samples are refrigerated at 4 °C. Samples are analysed within one week of collection if collected July-August, or if collected outside of July-August, frozen and analysed within 90 days.

PO_4_^3−^ is measured colorimetrically by the molybdenum blue method^21^ on a Lachat flow injection analyser (Hach, Loveland, CO). The method was modified for flow injection analysis by Lachat Instruments^22^. Samples are analysed within one week of collection if collected July-August, or if collected outside of July-August, frozen and analysed within 90 days.

TDN is analysed by persulphate digestion^23^ that oxidizes dissolved nitrogen to NO_3_^−^ and subsequent analysis of NO_3_^−^ by colorimetry on a Lachat flow injection analyser. To reduce the magnitude of the reagent blank, persulphate is recrystallized prior to analysis. Samples are stored at 4 °C prior to analysis and are analysed within two weeks of collection if collected July-August, or collected outside of July-August, frozen and analysed within 90 days.

Chl *a* and Pheo are measured with method of Arar *et al*.^24^ by filtering a known volume of water through a 25 mm glass fiber filter (GFF). On the day of sample collection, samples are filtered under low vacuum pressure (<10 psi) in dim light. Filters are then stored in 15 mL centrifuge tubes, in the dark at −20 °C until analysis. Filters are extracted using 7 mL of 90% buffered acetone followed by acidification. Optical filters are used to determine the excitation (420 nm) and emission (670 nm) wavelengths. Extracts are measured on a fluorometer (Turner Designs Model # 10-AU-005-CE). Chl *a* is calculated from the decrease in fluorescence caused by acidification. Pheo is calculated from the residual fluorescence after accounting for fluorescence by Chl *a*. The fluorometer is calibrated within one month of the first sampling date using solutions of pure Chl *a* of known concentrations. A solid secondary standard is used to check the instrument during the summer sampling season. Samples are analysed within two weeks if collected during July-August, or if collected outside of July-August, analysed within five weeks.

PON and POC are analysed by filtering through pre-combusted 25 mm GFF filters, recording filtered volume, drying at 65 °C, and combusting filters in a Perkin-Elmer 2400 or Thermo Flash 2000 elemental analyser^25^. Sample filters were not fumed with acid to remove any inorganic carbon. Samples are analysed within two weeks if collected during July-August, or if collected outside of July-August, stored dry and analysed within 120 days.

TP is analysed by persulphate digestion of unfiltered water samples following the method of Gales *et al*.^26^ and analysis of PO_4_^3−^ in the digest by colorimetry on the Lachat flow injection analyser. Samples are acidified to pH 2 with ultrapure hydrochloric acid (12 N) on the day of collection and analysed within two weeks if collected during July-August, or if collected outside of July-August, frozen and analysed within 90 days.

DOC was analysed by two methods: (1) catalytic oxidation and measurement of the combustion products on a Shimadzu TOC-V (2009 to 2014) and (2) persulphate oxidation on an Aurora 1030 W (2015 to 2018). Samples are acidified to pH <3 with phosphoric acid and stored at 4 °C. During analysis, inorganic carbon is removed by acidification and sparging of the sample with compressed ultra pure nitrogen gas^27^. Consensus Reference Material was used for sample years 2009 to 2012. In subsequent years, standards were prepared from dried potassium hydrogen phthalate to encompass the range of sample values, about 150 to 2,000 µM C.

## Data Records

The data are stored in a Microsoft Excel file. Each row in the data table represents a unique sample and the columns include values for different analytes, and quality assurance notes specific to that observation (Online-only Table 1). Metadata, station latitude and longitude, methods information, and a change log are stored as separate tabs in the Excel file. The data, metadata, and QAPPs may be obtained by contacting the Buzzards Bay Coalition. The data and metadata are available through the Woods Hole Open Access Server^28^.

## Technical Validation

Data were collected according to a series of Quality Assurance Project Plans (QAPPs) approved by the U.S. Environmental Protection Agency (EPA) and the Massachusetts Department of Environmental Protection (MassDEP). The first QAPP^29^ was approved in 1994 and updated versions approved in 1996^30^, 2001^31^, 2006^32^, 2009^33^ and 2014^14^. The QAPP covers both the field and laboratory components and details training requirements, data quality objectives such as laboratory precision, maximum hold times, reporting limits, sample handling protocols, documentation procedures, and quality control requirements. The MassDEP has accepted the data for determinations of classification of impaired waters under Clean Water Act regulations and the EPA has cited *Baywatchers* data as justifications for nitrogen effluent limits in permits for wastewater discharges to Buzzards Bay.

### Equipment calibration

At the beginning of each monitoring season, BBC staff train monitors (including returning monitors) in sample collection and analysis procedures and accurate data reporting. Monitors are given standardized handbooks that are updated each year. Monitor equipment (thermometers, hydrometers, Secchi disks, etc.) is examined and calibrated before the beginning of each season and checked at end of the season. Data are reviewed as monitors submit it during the season allowing for issuance of replacement equipment if the thermometers or hydrometers are suspected to be out of calibration based on unusual values. For D.O. analysis, new chemicals are ordered each year. YSI sondes are calibrated annually.

For each laboratory chemical assay, a complete standard curve is generated for each analytical run with a minimum of five points covering the range of sample concentrations. Primary standards are prepared at the beginning of each season and preserved with chloroform. Sequences of standards are prepared new each day from the primary standard. In addition, reagent blanks and new standards are prepared and analysed with new reagents for each sampling date. Least squares linear regression is used to calculate the standard curves for each assay and a minimum r^2^ = 0.99 is required for acceptance.

### Laboratory replicates

NH_4_^+^, NO_3_^−^ + NO_2_^−^, TDN, PO_4_^3−^, and TP are assayed in laboratory duplicates at a frequency of more than 10% of the samples and with a <10% tolerance between duplicates required for acceptance. For the particulate analyses (PON, POC, Chl *a*, Pheo), only field duplicates and laboratory standards can be assayed because the analysis consumes the entire sample. Spiked samples are run periodically as analytical checks for NO_3_^−^ + NO_2_^−^, NH_4_^+^, TDN, and PO_4_^3−^. Paired NO_3_^−^ and NO_2_^−^ standards are included in the beginning and end of each analytical run as checks on efficiency of NO_3_^−^ reduction to NO_2_^−^. No spikes are run for PON, but PON/POC standards of known concentration are added directly to filters as checks. Laboratory duplicates were highly repeatable (Table 1).

**Table 1:** Average relative standard deviation (RSD) and R^2^ for linear correlation of paired laboratory duplicate samples from 2017 and 2018.

Analyte	Average RSD (%)		R^2^	
	2017	2018	2017	2018
Ammonium	8.2	4.5	0.9969	0.9994
Nitrate + nitrite	8.8	6.0	0.9999	0.9999
Total dissolved N	9.7	7.8	0.8950	0.9733
Phosphate	4.5	6.9	0.9973	0.9931
Total phosphorus	6.0	4.8	0.9864	0.9885

### Field duplicates

**Fig. 4:**
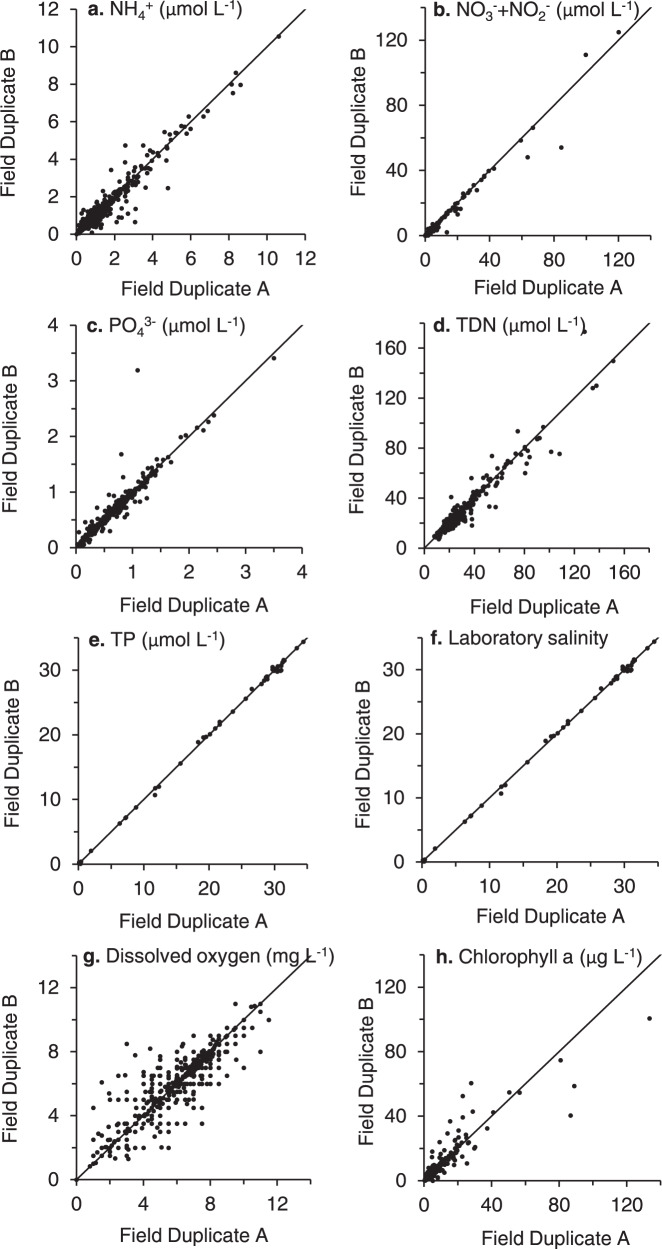
Comparison of *Baywatchers* field duplicate samples. Lines represent 1:1.

On one date each year, monitors collect and analyse field duplicate samples for D.O. During training at the beginning of the season, monitors are asked to also perform a duplicate D.O. measurement when they experience an unusual or very low D.O. test result and to alert BBC staff of the result. Field duplicate samples for laboratory analysis are collected 5% of the time and analysed within each analytical run. Comparison of field duplicates (Fig. 4) indicates good agreement between duplicates. The modified Winkler titration can take about 30 minutes to perform, so D.O. duplicates are often collected 30 to 45 minutes apart. Depending on the sampling location and the point in the tidal cycle there can be significant changes in the D.O. concentration between when the two duplicates are collected.

### Quality control review of data

Volunteers are asked to submit all data sheets every two weeks and the data are examined and entered as they are received. If unexpected values occur, volunteers are contacted and the results and monitoring procedures discussed and volunteers re-trained if necessary. At the end of each monitoring season, the D.O. data are again reviewed by BBC staff to identify any unusual observations or large changes from previous years. In December of each year, the laboratory chemistry data are reviewed by both the analytical laboratory staff and BBC staff. Unusual data are flagged for further review of the original data sheets and the laboratory data. Based on the review and discussions between analytical laboratory and BBC staff, the values are either accepted or rejected. In 2014, specific quality control flags were developed and implemented in the data file so that a record of any QC corrections is maintained as an integral part of the data file.

### Data comparison with other laboratories

Comparisons of *Baywatchers* data with two other datasets collected by other researchers during July and August demonstrate the data broadly represent the environments sampled. These comparisons were:

#### West Falmouth Harbor

Sampling of West Falmouth Harbor was initiated as part of the Falmouth Pond Watchers program in 1987 and continues to the present with laboratory analysis by the School for Marine Science and Technology, University of Massachusetts Dartmouth. *Baywatchers* began sampling 7 common stations (*Baywatchers* stations WF1N, WF2, WF4N, WF5N, WF6, WF9N, WF11) in 2013, although not necessarily on the same day.

#### Nantucket Sound

In 2012, *Baywatchers* and the Center for Coastal Studies (CCS) in Provincetown, MA both began sampling a common station in Nantucket Sound (*Baywatchers* station NTKS10). CCS analysed the samples for NO_3_^−^ + NO_2_^−^, NH_4_^+^, and PO_4_^3−^, but initially not for TDN, though CCS subsequently began analysing samples for TDN. In total, there were 84 overlapping station-months in estuarine waters between *Baywatchers* and SMAST (73) and between *Baywatchers* and CCS (11) for NH_4_, NO_3_^−^ + NO_2_^−^, and PO_4_ and 66 overlapping station-months between *Baywatchers* and SMAST for TDN and 1 overlapping station-month between *Baywatchers* and CCS for TDN. There was good agreement with the values measured by *Baywatchers* and the other programs with no statistical difference between the monthly averages 82% to 100% of the time (Table 2). The samples were collected at the same stations but not on the same dates, so some variability between labs would be expected based on actual temporal variability in concentrations. The similarity of *Baywatchers* data with other independent sampling of the same places provides confidence that *Baywatchers* data accurately reflect the water quality of the places sampled.

**Table 2:** Comparison of monthly averages of NH_4_^+^, NO_3_^−^ + NO_2_^−^, PO_4_^3−^, and TDN analysed by *Baywatchers*, the School for Marine Science and Technology (SMAST) at the University of Massachusetts, Dartmouth, and by the Center for Coastal Studies (CCS).

	NH_4_^+^	NO_3_^−^+NO_2_^−^	PO_4_^3−^	TDN
Estuarine Stations				
Total overlapping station-months	84	84	84	67
Station months where BBC & SMAST averages different	5 (7%)	1 (1%)	7 (10%)	2 (3%)
Station months where BBC & CCS averages different	2 (18%)	1 (9%)	1 (9%)	0 (0%)

Data were collected at the same stations by the separate labs not on the same dates but all at the end of ebb tide. Only months where both labs collected at least two samples were considered. Station-months with different averages were determined using Student’s t-test. There was only 1 overlapping station-month where CCS measured TDN.

## Usage Notes

Data collection will continue annually and each year an updated version of the dataset will be released. The metadata file contains a change log where any changes from the previous version are recorded. A small number of anomalous data results were excluded. The values are absent from the database and the exclusions can be identified by the values “8” or “9” in the corresponding QC columns. Data exclusions were rare, being only 0.1% of measurements.

Many stations in the database were sampled relatively briefly. Occasionally these were test stations for specific purposes. However, many of the stations that were sampled briefly are variants of regular stations that were collected because of logistical reasons (e.g., if a boat sampling station could not be accessed because of weather or boat malfunction, a substitute station might be a nearby dock). Because of the desire to combine alternative sampling locations like these, a name field was added to the database called Stn_Equiv. This field has the same station name as the Stn_ID field, except for alternate stations, where the Stn_Equiv field is assigned the station name of the regular station.

**Fig. 5:**
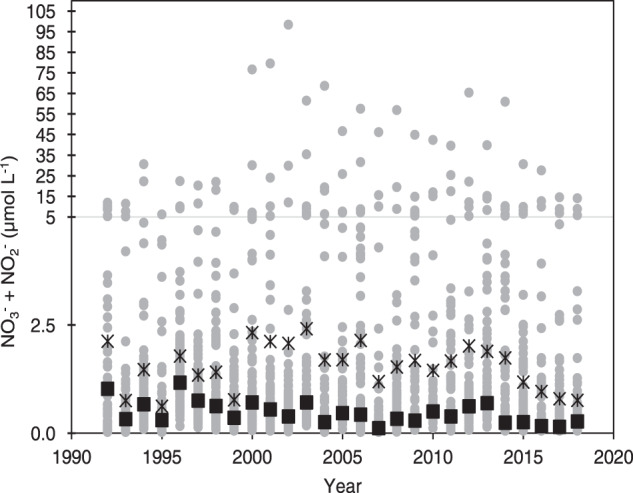
Annual average NO_3_^-^ + NO_2_^-^ concentrations of individual stations (gray dots). Buzzards Bay-wide average of NO_3_^-^ + NO_2_^-^ concentrations across all stations (black asterisks), and Bay-wide median NO_3_^-^ + NO_2_^-^ concentrations across all stations (black squares). Only stations for which there are 24 years or more of data included. Note the change in the y-axis scale at 5 μmol L^-1^.

Users of the data should be aware that there is wide range in the nutrient concentrations observed between sub-estuaries. While the majority of the stations have annual average NO_3_^−^ + NO_2_^−^ concentrations that are 1 µmol L^−1^ or less, the annual average of some locations can reach as high as 98 µmol L^−1^, particularly in areas of freshwater inflows (Fig. 5).

## Online-only Table

**Online-only Table 1:** *Baywatchers* water quality dataset variables and description. Each variable has a separate associated quality control note variable.

Variable	Description
STN_ID	Station identification code. Station coordinates are included on the “Stations” tab of the Excel file.
STN_EQUIV	Equivalent station name for temporary station variants or for stations paired by the Buzzards Bay Coalition for health index calculations.
SAMP_DATE	Date sample collected in mm/dd/yyyy format.
UNIQUE_ID	Unique identification code for each sample
SOURCE	Program Source (BBC = Coalition program, Pondwatchers = Falmouth Pond watchers, or other specific study)
LAB	Laboratory that performed laboratory chemical analyses: WHOI = Woods Hole Oceanographic Institution; SMAST = School for Marine Science and Technology; MBL = Marine Biological Laboratory.
GEN_QC	General quality control. A 9 in this column indicates all values for this sample were discarded.
TIME	Time sample collected in hh:mm am format.
TIME_QC	Quality control notes for time value.
DUP	“1”indicates that a field duplicate sample was collected. “0” indicates no duplicate was collected. “2” indicates the values in this row are the averages for paired duplicate samples.
SAMPDEP_M	Sample depth below the water surface in meters.
SAMPDEP_QC	Quality control notes for sample depth value.
SECCHI_M	Secchi depth in meters.
SECC_QC	Quality control notes for Secchi depth value.
TOTDEP_M	Total depth at time of sampling in meters.
TOTDEP_QC	Quality control notes for total depth value.
TEMP_C	Temperature in degrees Celsius.
TEMP_QC	Quality control notes for temperature value.
SAL_FIELD	Salinity measured in the field.
SAL_METH	Method used for salinity analysis. S1 indicates use of a hydrometer and thermometer. S2 means a water quality sonde was used. S3 indicates a refractometer was used.
SAL_QC	Quality control notes for salinity value.
SAL_LAB	Salinity measured in the laboratory on subsamples.
SAL_LAB_QC	Quality control notes on laboratory salinity value.
COND	Conductivity measured in the laboratory on subsamples.
DO_MGL	Dissolved oxygen in mg L^−1^.
DO_SAT	Dissolved oxygen percent saturation.
DO_METH	Method used for dissolved oxygen analysis. DO1 represents the modified Winkler titration method. DO2 indicates a water quality sonde was used.
DO_QC	Quality control notes for dissolved oxygen value.
PO4_UM	Soluble reactive phosphate concentration in μmol phosphorus L^−1^.
PO4_QC	Quality control notes for soluble reactive phosphate value.
TP_UM	Total phosphorus concentration in μmol L^−1^.
TP_QC	Quality control notes for total phosphorus value.
NH4_UM	Ammonium concentration in μmol nitrogen L^−1^.
NH4_QC	Quality control notes for ammonium value.
NO2NO3_UM	Nitrate + nitrite concentration in μmol nitrogen L^−1^.
NO2NO3_QC	Quality control notes for nitrate + nitrite value.
TDN_UM	Total dissolved nitrogen concentration in μmol nitrogen L^−1^.
TDN_QC	Quality control notes for total dissolved nitrogen value.
PON_UM	Particulate organic nitrogen concentration in μmol nitrogen L^−1^.
PON_QC	Quality control notes for particulate organic nitrogen.
TN_UM	Total nitrogen concentration in μmol nitrogen L^−1^.
TN_QC	Quality control notes for total nitrogen.
POC_UM	Particulate organic carbon concentration in μmol carbon L^−1^.
POC_QC	Quality control notes for particulate organic carbon.
CHLA_UGL_LAB	Chlorophyll *a* concentration in μg L^−1^ measured in a laboratory.
CHLA_LAB_QC	Quality control notes for chlorophyll *a* value.
CHLA_LAB_METH	Method used for chlorophyll *a* analysis. CH1 represents the acetone extraction method. CH2 indicates a water quality sonde was used.
CHLA_UGL_SONDE	Chlorophyll *a* concentration in μg L^−1^ measured using a water quality sonde.
CHLA_ SONDE_QC	Quality control notes for chlorophyll *a* value.
CHLA_SONDE_METH	Method used for chlorophyll *a* analysis. CH1 represents the acetone extraction method. CH2 indicates a water quality sonde was used.
PHEO_UGL	Pheophytin *a* concentration in μg L^−1^.
PHEO_QC	Quality control notes for pheophytin *a*.
DOC_UM	Dissolved organic carbon concentration in μmol L^−1^.
DOC_QC	Quality control notes for dissolved organic carbon concentration value.
PH	pH measured by water quality sonde.
PH_QC	Quality control notes for pH value.
BEAU	Beaufort scale value as observed by monitors.
BEAU_QC	Quality control notes for Beaufort scale value.
TIDE	Tide direction recorded by monitors. E indicates sample collected on ebb tide, F indicates sample collected on flood tide.
WHTR	Weather observations made by monitors based on the following scale: 1 = cloudless, 2 = partly cloudy, 3 = overcast, 4 = fog/haze, 5 = drizzle, 6 = intermittent rain, 7 = rain, 8 = snow.
PREC	Precipitation in the past 24 hours recorded by monitors as either 1 = none, 2 = light, or 3 = heavy.
WIND	Wind direction as estimated by monitors.
OBS_COMM	Observations made by monitors.
QA_COM	Other quality control notes made by *Baywatchers* staff.

Quality control note codes are: 1 = Centigrade recalculated from Fahrenheit; 2 = Value needs to be reviewed for accuracy; 3 = Value reviewed then confirmed, rerun, or revised; 4 = Flagged as unusual value, with comments about possible reasons if known; 6 = Substitute value with comment for details; 7 = Data below method detection limit.; 8 = Value highly anomalous, although laboratory measurements were validated, sample may have been compromised during collection; 9 = Value rejected; 10 = Laboratory salinity substituted for missing field salinity; 11 = Field salinity restored to original value for 1992–1995 for samples that had a correction factor applied at the time; 12 = Time of D.O. collection too late for D.O. value to be used in Buzzards Bay Coalition health index calculations. Column QA_COM includes other general quality control comments.
